# eHealth Trends in Europe 2005-2007: A Population-Based Survey

**DOI:** 10.2196/jmir.1023

**Published:** 2008-11-17

**Authors:** Per Egil Kummervold, Catherine E Chronaki, Berthold Lausen, Hans-Ulrich Prokosch, Janne Rasmussen, Silvina Santana, Andrzej Staniszewski, Silje Camilla Wangberg

**Affiliations:** ^7^Department of Family MedicineWrocław Medical UniversityWrocławPoland; ^6^DEGEI/IEETAUniversity of AveiroAveiroPortugal; ^5^MedCom InternationalMedComOdenseDenmark; ^4^Department of Medical InformaticsBiometrics and EpidemiologyUnit for Medical InformaticsErlangenGermany; ^3^Department of Medical InformaticsBiometrics and EpidemiologyUnit for Biometrics and EpidemiologyErlangenGermany; ^2^Institute of Computer ScienceFoundation for Research and Technology-HellasHeraklionCreteGreece; ^1^Norwegian Centre for TelemedicineUniversity Hospital of North-NorwayTromsøNorway

**Keywords:** Internet, patient-provider communication, Internet health communication, electronic mail, information services, trends, and utilization, medical informatics, health services, demography, data collection, health care surveys

## Abstract

**Background:**

In the last decade, the number of Internet users worldwide has dramatically increased. People are using the Internet for various health-related purposes. It is important to monitor such use as it may have an impact on the individual’s health and behavior, patient-practitioner roles, and on general health care provision.

**Objectives:**

This study investigates trends and patterns of European health-related Internet use over a period of 18 months. The main study objective was to estimate the change in the proportion of the population using the Internet for health purposes, and the importance of the Internet as a source of health information compared to more traditional sources.

**Methods:**

The survey data were collected through computer-assisted telephone interviews. A representative sample (N = 14,956) from seven European countries has been used: Denmark, Germany, Greece, Latvia, Norway, Poland, and Portugal. The European eHealth Consumer Trends Survey was first conducted in October-November 2005 and repeated in April-May 2007. In addition to providing background information, respondents were asked to rate the importance of various sources of health information. They were also queried as to the frequency of different online activities related to health and illness and the effects of such use on their disposition.

**Results:**

The percentage of the population that has used the Internet for health purposes increased from an estimated 42.3% (95% CI [Confidence Interval] 41.3 - 43.3) in 2005 to an estimated 52.2% (95% CI 51.3 - 53.2) in 2007. Significant growth in the use of the Internet for health purposes was found in all the seven countries. Young women are the most active Internet health users. The importance of the Internet as a source of health information has increased. In 2007, the Internet was perceived as an important source of health information by an estimated 46.8% (95% CI 45.7 - 47.9) of the population, a significant increase of 6.5 % (95% CI 4.9 - 8.1) from 2005. The importance of all the traditional health information channels has either decreased or remained the same. An estimated 22.7% (95% CI 21.7 - 23.6) are using it for more interactive services than just reading health information.

**Conclusion:**

The Internet is increasingly being used as a source of health information by the European population, and its perceived importance is rising. Use of the Internet for health purposes is growing in all age groups and for both men and women, with especially strong growth among young women. We see that experienced Internet health users are also using the Internet as an active communication channel, both for reaching health professionals and for communicating with peers.

## Introduction

In recent years, the number of Internet users has increased considerably and the Internet is being used for various health purposes [1-5]. Health professionals, patient organizations, and the pharmaceutical industry are using the Internet as a medium for communicating health information [6-9]. For patients the most obvious use is as a source of health information. Nevertheless, we also see that they use it for accessing and managing their own personal health record [10-13], for purchasing health products and services, and for communicating with peers and health professionals [14,15].

However, most of the literature on this issue comes from the United States. Studies on the use of Internet-based technologies for health purposes within Europe are still rare [16,17]. To chart the European status of this development, a survey in Denmark, Germany, Greece, Latvia, Norway, Poland, and Portugal was conducted in 2005 as the first phase of the WHO eHealth Consumer Trends Survey funded by the European Commission. This baseline study showed that even if there were considerable regional differences, a significant proportion of citizens in all the countries studied were using Internet for health purposes [18-20].

To study the pace and direction of the European citizens’ appraisal of eHealth services, we repeated the survey in 2007. In addition to studying trends in the general population, the study also focuses on whether the type and frequency of health-related activities on the Internet change as the medium matures and Internet users become more experienced. In doing so, we hope to shed some light on the future of Internet-based services for health and illness.

## Methods

### Participants and Procedure

The first survey was conducted in the period from October to November 2005. Random digit dialling in strata was used to ensure a randomized representative sample of the seven participating countries. Sampling continued until we had approximately 1000 completed interviews per country, except for Portugal where the limit was increased to 2000 complete interviews, as health-related Internet use was expected to be low.

The second survey took place in April and May 2007. Experiences from the first survey showed that the sample was skewed for some age groups. In 2007, quotas were therefore constructed based on census data for age and gender to make sure the data were more representative in this regard. This ensured that the sample had the same distribution age (six groups) and gender as the census. As described below, weighting were used on the 2005 data to adjust them accordingly. The target sample size was set to 1000 for all countries in 2007.

Mobile phone numbers were included in Norway, Denmark, and Latvia. In the other countries only landline telephones were included since it was difficult to get a reliable sample based on mobile phones in these countries in 2005. In the countries where mobile phones were included, the telephone penetration was close to 100% for Norway and Denmark, while it was around 93% in Latvia. In the countries where only landline telephones were used, the telephone penetration was estimated for 2005/2007 to be 87/82% in Greece, 63/64% in Poland, and 65/60% in Portugal. In Germany is was close to 100%.

To get a response rate for telephone interviews comparable to ordinary interviews can be challenging. In 2005, we were not able to get comparable numbers from all countries allowing us to give an exact response rate. This procedure was improved for 2007. Problems reaching the target person can be divided into two groups. The first is “no contact”, including incorrect numbers, disconnected numbers, and answering machines. When we are doing stratified sampling with no additional details about the person we are calling, this group also contains people not in the target group. This group was on average 58% of the total numbers called (min: 36% in Germany, max: 76% in Denmark). If we want a response rate comparable to ordinary interviews, it is reasonable to exclude this group. We should then calculate the response rate of the people that actually had a chance to participate.

The second group is “non-responses”, including people not wanting to participate in interviews, people not having time to participate, language problems, interrupted interviews, and people being too sick to participate. Using this number for calculation, we get an average response rate of 36% (min: 17% in Greece, max: 60% in Latvia).

National ethics committees in all the participating countries were informed and had no objections to the study. The data was analysed using the SPSS software version 15.0 and R version 2.5.1.

### Measures

The questionnaire used in the study was designed for computer-assisted telephone interviews (CATI). The questionnaire was first designed in English. A dual-focus approach was then used for translating it into the languages of the seven European countries participating in the survey: Denmark, Germany, Greece, Latvia, Norway, Poland, and Portugal. The dual-focus approach strives for conceptual equivalence rather than wording and grammar, and is a modification of the back-translation method [21]. After the translation, the questionnaire was piloted with 100 individuals in Norway.

Internet use for health purposes was measured with the question, “How often do you use the Internet to get information about health or illness?”. The response alternatives were: “Every day”, “Every week”, “Every month”, “Every six months”, “Every year”, “Less than once a year”, and “Never”. All not answering “Never” were coded as having used the Internet for health purposes. To measure the importance of different health information channels, the respondents were asked to rate their importance on a scale going from 1 “not important” to 5 “important”.

### Data Analyses

Mainly the analyses compare change in proportion from 2005 to 2007. Secondly, differences in proportions by demographic variables such as age and gender are assessed. Significant change is judged by non-overlapping confidence intervals (CI). All reported CI’s are 95%. The CIs are derived by Gaussian approximations of the distribution of the sum of strata frequencies or sum of ratios of strata frequencies. *P*-values of two sided tests are not given. Significant test results are reported when the null is not inside the 95% interval.

The 2005 data were weighted based on the 2007 distribution regarding age and gender. The reason for weighting the data was to distinguish real effects from minor changes in the demographics of the samples. The weighting also corrected for differing sample sizes, mainly Portugal in 2005. Unfortunately, the weighting means that it would be confusing to state absolute numbers of respondents to each question for 2005.

All countries contribute equally to the grand total, but numbers weighted for population size are also stated in Table 1.

Logistic regression analysis was calculated using employment status, year, gender, and age as dependent variables for the independent variables Internet user, Internet health users, and users of interactive Internet health services. For each variable, we report odds ratios and 95% confidence intervals of the odds ratios.

## Results

### General Trends

On average, the percentage of the population that had used the Internet for health purposes increased from 42.3% (41.3 - 43.3)in 2005 to 52.2% (51.3 - 53.2) in 2007 (Table 1). There were regional differences. The lowest 2007 use was registered in Greece at 32.1% (29.5 - 34.7) and Portugal at 38.3% (35.6 - 41.0). The highest use was recorded in Denmark at 71.6% (69.1 - 74.1) and Norway at 66.8% (64.2 - 69.5) [22].

Significant growth in the use of Internet for health purposes was found in all the seven countries participating in the survey, with an average growth of 9.9% (8.5 - 11.3). Highest growth was noted in Germany (12.2%), Poland (11.8%), and Latvia (11.3%), whereas the lowest growth was noted in Portugal (9.1%), Greece (8.9%), and Norway (6.6%).

**Table 1:** Internet health users in the seven European countries—trends 2005 and 2007 (an expanded version that also includes Internet users is available as Multimedia Appendix 1)

			Internet health users		
	Pop. Weight	2005/2007	2005	2007	Growth
Country		Count (N)	% (CI)	% (CI)	% (CI)
Denmark	3,5	960/1021	61.8 (59.0 - 64.7)	71.6 (69.1 - 74.1)	9.8 (6.0 - 13.6)
Germany	53,4	974/1000	44.4 (41.4 - 47.5)	56.6 (53.9 - 59.3)	12.2 (8.1 - 16.2)
Greece	7,2	1000/1000	23.2 (20.7 - 25.7)	32.1 (29.5 - 34.7)	8.9 (5.3 - 12.5)
Latvia	1,5	1000/1000	35.7 (33.2 - 38.2)	47.0 (44.4 - 49.6)	11.3 (7.7 - 14.9)
Norway	3,0	972/1001	60.3 (57.4 - 63.1)	66.8 (64.2 - 69.5)	6.6 (2.7 - 10.4)
Poland	24,7	1027/1000	41.5 (38.8 - 44.2)	53.3 (50.6 - 56.0)	11.8 (8.0 - 15.6)
Portugal	6,8	2001/1000	29.2 (27.4 - 31.1)	38.3 (35.6 - 41.0)	9.1 (5.8 - 12.3)
Average			42.3 (41.3 - 43.3)	52.2 (51.3 - 53.2)	9.9 (8.5 - 11.3)
Average (weighted for population size) (See note under Methods)			42.1 (40.3 - 43.9)	53.5 (51.9 - 55.1)	11.4 (9.0 - 13.7)

### Demographics

**Figure 1:**
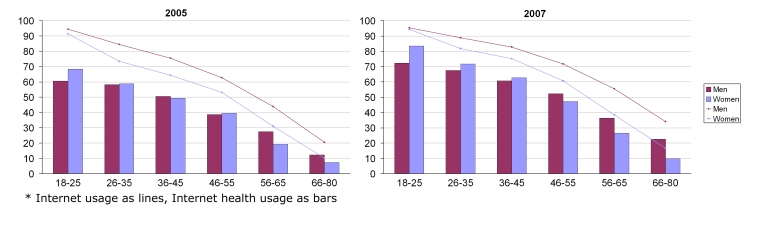
Internet and Internet health usage in 2005 and 2007, by age and gender (numbers are available in Multimedia Appendices 2 and 3)

In 2005, there were significantly more men using the Internet in all age groups. This difference seems to have diminished and was no longer significant in 2007 for the youngest age group (15 - 25 years). Of women aged 15 - 25 years, 83.5% used the Internet for health purposes in 2007. The corresponding proportion for men was 72.4%. At the other end of the age scale (66 - 80 years), we saw the opposite effect, where 22.6% of men and 9.9% of women used the Internet for health purposes (Figure 1, Multimedia Appendix 3). The same effect was visible in 2005, but it was not so clear.

### The Internet as a Source of Health Information

The participants rated the importance of various sources of health information on a scale from 1 to 5. The top two alternatives (4 = important and 5 = very important) were recoded as “important” in Table 2. The Internet had a 6.5% (5.0 - 8.1) increase, and in 2007 was characterized as important by 46.8% (45.7 - 47.9) of the population. Direct contact with health professionals, although decreasing from 2005, was still perceived as the most important source of health information with 73.8% (72.8 - 74.8) describing it as important. It was followed by “family, friends, and colleagues” at 63.8% (62.8 - 64.9). The sharpest decline was observed in newspapers and magazines, which had a 5.1% (3.5 - 6.7) decrease to 48.2% (47.0 - 49.3).

**Table 2:** Importance of various sources for health information

	2005	2007	
	N = 7934	N = 7022	Change
	Mean % (CI)^a^	Frequency Mean % (CI)	Mean Difference % (CI)
Health professionals	77.5 (76.5 - 78.5)	5180 73.8 (72.8 - 74.8)	-3.7 (-5.1 - -2.3)
Family, friends, and colleagues	63.6 (62.5 - 64.7)	4480 63.8 (62.8 - 64.9)	+0.3 (-1.3 - 1.8)
TV/radio	57.1 (56.0 - 58.2)	3763 53.6 (52.5 - 54.8)	-3.5 (-5.1 - -1.9)
Pharmacies	55.4 (54.3 - 56.5)	3646 52 (50.8 - 53.1)	-3.4 (-5.0 - -1.8)
Newspapers and magazines	53.3 (52.2 - 54.4)	3380 48.2 (47.0 - 49.3)	-5.1 (-6.7 - -3.5)
Books	51.6 (50.5 - 52.7)	3353 47.8 (46.7 - 48.9)	-3.8 (-5.3 - -2.2)
Internet	40.3 (39.2 - 41.4)	3288 46.8 (45.7 - 47.9)	+6.5 (5.0 - 8.1)
Courses and lectures	32.9 (31.8 - 33.9)	2249 32.1 (31.0 - 33.1)	-0.8 (-2.3 - 0.7)

^a^ 2005 data were weighted based on the 2007 distribution regarding age and gender. Absolute numbers are therefore not reported

There is considerable variation in the importance placed on the Internet as a source of health information within the seven European countries studied. In Denmark currently, the Internet is already considered the second most important source, preceded only by “health professionals”. At the other end of the scale, in Greece, the Internet is considered the least important source of information about health and health-related problems (Multimedia Appendix 4). All countries do, however, show significant growth in the importance placed on the Internet, with the exception of Germany where the increase is not significant.

### Usage Patterns

The percentage of consumers using the Internet for health purposes in other, more interactive, ways did increase from 15.3% (14.5 - 16.1) in 2005 to 22.7% (21.7 - 23.6) in 2007 (Table 3). In 2007 a total of 9.9% (9.2 - 10.6) have participated in health related forums or self-help activities more than once a year. The study also shows that 8.5% (7.8 - 9.1) order medical health products online, 11.1% (10.4 - 11.8) have online communication with health professionals whom they have not previously met, and 6.9% (6.3 - 7.4) have used the Internet to interact with known health professionals. The use of all interactive, health-related online services increased significantly. Multimedia Appendix 5 also includes the numbers for the subsamples of Internet users and Internet health users.

**Table 3:** Percentage of consumers who are using interactive Internet health services at least once a year (Multimedia Appendix 5 shows the percentages for Internet users and Internet health users)

	2005	2007	Change
	Mean % (CI)^a^	Frequency (n) Mean % (CI)	Mean % (CI)
Self-help activities	7.0 (6.4 - 7.6)	694 9.9 (9.2 - 10.6)	2.9 (2.0 - 3.8)
Order medicine or other health products	5.5 (4.9 - 6.0)	596 8.5 (7.8 - 9.1)	3.0 (2.2 - 3.9)
Interact with Web doctor/health professional you have not met	8.2 (7.6 - 8.8)	780 11.1 (10.4 - 11.8)	2.9 (2.0 - 3.9)
Approach family doctor or other known health professional	3.6 (3.2 - 4.1)	484 6.9 (6.3 - 7.4)	3.2 (2.5 - 4.0)
Using at least one of the interactive services above	15.3 (14.5 - 16.1)	1593 22.7 (21.7 - 23.6)	7.4 (6.2 - 8.6)

^a^ 2005 data were weighted based on the 2007 distribution regarding age and gender. Absolute numbers are therefore not reported

From a country-specific point of view (Multimedia Appendix 6), we observed large increases in specific interactive activities in Denmark and Germany. In Denmark the percentage of Internet users who approach a family doctor or other known health professional online has increased by 12.2% (9.1 - 15.2) to 20.1% (17.6 - 22.6) in 2007. In Germany the percentage of Internet users ordering medicine or other health products has increased by 6.2% (3.1 - 9.3) to 17.7% (15.3 - 20.1) in 2007.

Using logistic regression models (Table 4), we analysed trends from 2005 to 2007, looking at age, gender, and employment status and their effect on the use of the Internet, Internet health information, and interactive health services. The logistic analysis shows no significant effect of gender on the use of the Internet. There is, however, a significant interaction effect between gender and age, where the proportion of men is largest in the highest age groups. Employment status is also a significant factor, since a very large proportion of students are using the Internet.

For the Internet health user, the gender difference is much clearer, since women are using the Internet significantly more (OR = 2.92, 95% CI 2.36 - 3.62). Age seems to have less effect in predicting numbers of Internet health users than in predicting Internet users. There has been significant growth in the number of Internet users, Internet health users, and people using interactive Internet health services. There seems to be significant growth in the number of men using interactive health services as well.

**Table 4:** Factors affecting Internet usage, Internet health usage, and the use of interactive health services. Sample is based on all respondents in all seven countries (N = 14,955).

		Internet user	Internet health user	Interactive health user
		Odds ratio (95% CI), *P*	Odds ratio (95% CI), *P*	Odds ratio (95% CI), *P*
**Age**	10 year intervals^a^	0.61 (0.58 - 0.64), < .001	0.77 (0.74 - 0.81), < .001	0.80 (0.76 - 0.85), < .001
**Year**	2005	1	1	1
	2007	1.49 (1.10 - 2.01), .01	2.06 (1.66 - 2.57), < .001	1.77 (1.39 - 2.27), < .001
**Gender**	Male	1	1	1
	Female	1.15 (0.86 - 1.52), .35	2.92 (2.36 - 3.62), < .001	2.54 (1.99 - 3.23), < .001
**Employment**	Unemployed	1	1	1
	Work	3.51 (3.21 - 3.84), < .001	2.68 (2.46 - 2.92), < .001	1.67 (1.49 - 1.87), < .001
	Student	10.57 (7.913 - 14.12), < .001	2.83 (2.42 - 3.31), < .001	1.49 (1.26 - 1.77), < .001
**Interactions**				
	Age * Gender	0.90 (0.85 - 0.95), < .001	0.80 (0.76 - 0.83), < .001	0.835 (0.790 - 0.882), < .001
	Age * Year	1.04 (0.98 - 1.10), .18	0.97 (0.92 - 1.01), .14	1.04 (0.98 - 1.10), .21
	Gender * Year	0.93 (0.79 - 1.10), .39	1.02 (0.89 - 1.19), .75	0.82 (0.67 - 0.99), .03

^a^Odds Ratio (OR) is estimated for every 10 year difference

## Discussion

A majority of our European study population now uses the Internet for health purposes. We have seen a significant increase in all countries (Table 1).To a great extent this increase in use can be explained by improved Internet access.

In Denmark, Germany, Greece, and Portugal, we see that growth in the number of Internet health users is larger relatively speaking than growth in the number of Internet users. This might indicate that new Internet services for health users have been launched in these countries. In Denmark and Germany, our results also show a significant increase in one of the interactive health services. The relatively large increase in Internet users buying medicines online in Germany is matched by the growing eCommerce market for medicine since new legislation was introduced in 2004 [23,24]. In Denmark, we observe an increase in online communication with known health professionals as more and more GPs offer services to meet the expectations of their patients and to implement these services before January 2009, when it will become mandatory for GPs to offer online services [25].

### Demographics

There is still a majority of men representing Internet users in the seven countries studied. However, the difference between men and women is diminishing in younger age groups, and the 2007 survey did not show any significant difference between male and female Internet users for respondents aged between 15 and 25. Nearly all in this age group do have access to the Internet. It is therefore logical that it is among the oldest users that we have the largest growth potential.

The gender differences in Internet health use should be seen in the context of overall Internet use. Elderly people and women are traditionally overrepresented as health care receivers. This notion stands in contrast to the characteristics of the average Internet user. Internet health users are a combination of these factors. Looking at the youngest age group, we saw in 2005 that there were more women than men using Internet health services. The difference was 7.6% (4.6 - 10.7). In 2007, this difference increased to 11.1% (8.3 - 13.9). In other words, young women were already overrepresented as Internet health users in 2005, and it seems like this tendency increased in 2007.

In the logistic regression in Table 4, some interesting interaction effects can also be observed. While we can see an overall growth in Internet health usage among women, this does not apply to the oldest age group. Here the growth is largest among men, and it is not growth that can be explained by growth in general Internet usage. It is difficult to say why this is happening. One explanation might be that it is due to specific Internet services that target elderly men. Another might be that using the Internet for specific purposes like health is of greater interest to users in older age groups. The first adopters of the Internet in these groups were men, and perhaps they are now among those who use it for health purposes.

### The Internet as a Source of Health Information

The importance of the Internet as a source of health information is growing. The absolute numbers for this kind of Internet use in every country seem to rely on how the scale for ranging the importance of health information channels is interpreted. It seems more reliable to focus on the relative importance of the Internet as a health information source (compared to the traditional ones) in a specific country and on the change within that country from 2005 to 2007. From this perspective, it is interesting to notice that the greatest change in the importance of the Internet actually occurs in the countries that already had a high Internet health usage in 2005.

In Denmark for instance, the Internet was the second most important source of health information in 2007—outranked only by information from health professionals. Both in Norway and in Denmark, the Internet is now considered more important as a source of health information than television and radio. The aggregated results for all the countries show that, even if the Internet is at the bottom of the list, there is just a small, non-significant difference between the Internet and more traditional media such as books, newspapers, and magazines. As the latter media decrease in importance and the Internet increases, it is legitimate to predict that the Internet might surpass them over the next few years.

### Interactive Use of the Internet for Health Purposes

More in-depth analysis of the actual eHealth activities performed by the Internet health users in our two surveys reveals a tendency toward more “advanced” and more interactive use of the Internet for health purposes. Rather than using the Internet to search for and read health information, people are increasingly taking part in online communication with peers, unknown professionals, and their family doctors. In addition, the Internet is being used by more people for ordering medical health products.

Beckjord [5] estimates that 7% of US Internet users communicated online with a health care provider in 2003, and this increased to 10% in 2005. Since our study distinguishes between known health professionals and medical personnel whom patients meet only online, and since the study period is different, it is hard to make a direct comparison. Multimedia Appendix 5 extends Table 3 to give numbers for the Internet users only. This shows that interaction with Web doctors and other health professionals whom patients have not personally met did increase from 13.2% to 16.8% from 2005 to 2007. Communication with family doctors or other known health professionals increased from 5.0% to 9.7%. Even if the studies are not directly comparable, they give an indication that the average use in our seven European countries is not falling behind the average for the US.

However, both in the US and in Europe, it does seem that the overall trend is moving toward an increase in communication with health personnel over the Internet. The main factor driving this trend is patient demand for such services. In general it appears that patients are considerably more positive in their attitudes toward online communication than physicians are [26]. Other factors influencing this development are legislation, tariffs, and technical limitations. We see that the legislators are starting to take the consequences of this trend into account in Denmark, where all general practitioners will be obligated to offer eHealth-services to patients in 2009 [25].

We can, therefore, see a general growth in the use of the Internet for health purposes which parallels an increase in more interactive use. Even if our study does not follow the same individuals over time, it seems logical to assume that simply browsing for health information is the starting point. It seems that, when Internet users become more experienced and comfortable with opportunities provided by the Web, they also start to use it for two-way communication, either with peers in forums or with health professionals. This could be called the second generation of Internet health users, and the trend which we detect in Web use for health purposes parallels the current movement in general Internet use toward more interactive usage of the so-called Web 2.0.

Nevertheless, the Internet is still a relatively young medium, and its widespread use in some countries at present might still be limited by bandwidth and technical difficulties. We therefore expect that the proportion of more interactive Internet health use will grow significantly in the years to come. Additionally, health services which today still seem to be in a more or less premature phase, or are only recognized by a minority of the population, such as online access to one’s medical record from a health care provider or even managing one’s own personal health record, will gain in importance in the coming years.

With the current movement of mass software providers such as Microsoft and Google into the health care market [27], we will probably see a tendency among Internet health users to demand a more equitable role in their health care process. As stated by Ball and Lillis in 2001, new eHealth technologies provide opportunities for more empowered patients, and physicians need to be prepared for the likelihood that patients will start acting more as consumers [28] and challenge the current asymmetry of knowledge [29] in order to achieve a much fuller participation in health care decision-making processes.

### Limitations

The WHO eHealth consumer trends survey is based on previous surveys carried out in Norway as well as in Europe. Particular attention was devoted to the questionnaire addressing cultural differences with the dual-focus method and pilot surveys. Some variables turned out to be difficult to include in this comparison. One of them was education. There are seven independent educational systems in the countries studied. We used ISCED codes [30] in order to compare education across countries. The codes are fairly complicated to use, and we detected variations in how they were interpreted over the course of two years. We therefore decided to drop this variable in the analysis.

Another useful variable in the analysis would have been household income. In several of the countries in the study it would, however, have been inappropriate to ask about this in a telephone interview. Even if the question were included in the first Norwegian study, we would have had to drop it in the international questionnaire.

There was an interval of 18 months between the surveys. This is a fairly short time period, and many of the effects studied may not have been significant in such a short time span.

Another limitation of the study is the use of CATI and the sizable percentage of the population which cannot be reached using landline phones. The lack of public mobile-phone directories in several of the countries studied made it hard drawing representative samples. We used strata in compensation for this in 2007. Our main focus in this article is changes between 2005 and 2007. We were therefore especially concerned that such differences could be caused by demographic variation in the samples and chose to use weighting of the 2005 data, as described in Methods. This is not ideal, but we are confident that, in our situation, this did improve the quality of the analysis.

### Conclusion

The perceived importance of the Internet as a health information source is increasing. There is relative growth in all age groups and for both men and women in Internet use for health purposes, with especially strong growth among young women. Along with this growth, we also see that the second generation of Internet health users is using the Internet for more than just reading information. They are using the Internet as a channel, for direct communication with health professionals as well as with peers.

Our research has now been able to detect small trends over a two-year period. It will be important to follow up on this research in upcoming years and evaluate whether this trend in second-generation Internet health users continues. Physicians need to be aware of their patients’ use of such new technologies, since this might lead to much better informed patients and requests from patients for more interactive, Internet-based communication pathways.

## Multimedia Appendix 1

Internet users and Internet health users in the seven European countries—Trends 2005 and 2007

## Multimedia Appendix 2

Internet users by age and gender

## Multimedia Appendix 3

Internet health users by age and gender

## Multimedia Appendix 4

Importance of the Internet in various countries

## Multimedia Appendix 5

Expanded version of Table 3—percentage of consumers who are using interactive Internet health services at least once a year

## Multimedia Appendix 6

Total and estimated relative frequency of Internet health users and Internet users who are using at least one of the interactive services at least once a year

## Multimedia Appendix 7

Questionnaire in English (used as basis for translation in 2007)

## Abbreviations

CATI: computer-assisted telephone interviews
GP: general practitioner
ISCED: International Standard Classification of Education
WHO: World Health Organization
