# Supplementary material for: eHealth Trends in Europe 2005-2007: A Population-Based Survey
Source: J Med Internet Res. 2008 Nov 17;10(4):e42. doi: 10.2196/jmir.1023 (PMC2629359; doi:10.2196/jmir.1023)
Supplement: Supplementary file 1 [file jmir_v10i4e42_app1.pdf]

Multimedia Appendix 1. Internet users and Internet health users in the seven European countries—trends 2005 and 2007

			**Internet users**			**Internet health users**		
	**Pop. Weight**	**2005/2007**	**2005**	**2007**	**Growth**	**2005**	**2007**	**Growth**
**Country**		**Count (N)**	**% (CI)**	**% (CI)**	**% (CI)**	**% (CI)**	**% (CI)**	**% (CI)**
**Denmark**	3,5	960/1021	81.4 (79.3-83.5)	87.1 (85.3-88.8)	5.7 (2.9-8.4)	61.8 (59.0-64.7)	71.6 (69.1-74.1)	9.8 (6.0-13.6)
**Germany**	53,4	974/1000	63.3 (60.6-66.0)	64.9 (62.4-67.4)	1.6 (-2.0-5.3)	44.4 (41.4-47.5)	56.6 (53.9-59.3)	12.2 (8.1-16.2)
**Greece**	7,2	1000/1000	42.3 (39.7-45.0)	47.2 (44.7-49.7)	4.9 (1.2-8.5)	23.2 (20.7-25.7)	32.1 (29.5-34.7)	8.9 (5.3-12.5)
**Latvia**	1,5	1000/1000	53.8 (51.4-56.2)	66.7 (64.5-68.9)	12.9 (9.6-16.1)	35.7 (33.2-38.2)	47.0 (44.4-49.6)	11.3 (7.7-14.9)
**Norway**	3,0	972/1001	81.3 (79.3-83.2)	87.9 (86.2-89.6)	6.6 (4.1-9.2)	60.3 (57.4-63.1)	66.8 (64.2-69.5)	6.6 (2.7-10.4)
**Poland**	24,7	1027/1000	52.8 (50.3-55.4)	66.7 (64.3-69.1)	13.9 (10.4-17.4)	41.5 (38.8-44.2)	53.3 (50.6-56.0)	11.8 (8.0-15.6)
**Portugal**	6,8	2001/1000	49.0 (47.4-50.6)	52.3 (49.8-54.8)	3.3 (0.3-6.3)	29.2 (27.4-31.1)	38.3 (35.6-41.0)	9.1 (5.8-12.3)
**Average**			60.6 (59.7-61.4)	67.5 (66.7-68.4)	7.0 (5.7-8.2)	42.3 (41.3-43.3)	52.2 (51.3-53.2)	9.9 (8.5-11.3)
**Average (weighted for population size) (See note under Methods)**			59.3 (57.7-60.8)	64.7 (63.2-66.2)	5.4 (3.3-7.6)	42.1 (40.3-43.9)	53.5 (51.9-55.1)	11.4 (9.0-13.7)
