# Supplementary material for: eHealth Trends in Europe 2005-2007: A Population-Based Survey
Source: J Med Internet Res. 2008 Nov 17;10(4):e42. doi: 10.2196/jmir.1023 (PMC2629359; doi:10.2196/jmir.1023)
Supplement: Supplementary file 2 [file jmir_v10i4e42_app2.pdf]

Multimedia Appendix 2. Internet users by age and gender

	2005			2007		
	**Men % (CI)**	**Women % (CI)**	**Diff (CI)**	**Men Frequency/N % (CI)**	**Women Frequency/N % (CI)**	**Diff (CI)**
**15-25**	94.5 (93.4-95.6)	91.5 (90.4-92.7)	3.0 (1.4-4.6)	659/696<br>95.3 (94.3-96.4)	544/575<br>94.3 (93.3-95.2)	1.0 (-0.4-2.4)
**26-35**	84.6 (83.0-86.2)	73.5 (71.7-75.2)	11.1 (8.8-13.5)	524/598<br>88.8 (87.5-90.2)	450/557<br>81.8 (80.3-83.4)	7.0 (4.9-9.0)
**36-45**	75.5 (73.6-77.4)	64.3 (62.3-66.3)	11.1 (8.4-13.9)	526/631<br>82.8 (81.1-84.6)	510/679<br>75.2 (73.4-77.0)	7.6 (5.1-10.1)
**46-55**	62.8 (60.8-64.8)	53.2 (51.2-55.2)	9.6 (6.7-12.4)	388/533<br>71.8 (69.8-73.7)	372/611<br>60.7 (58.7-62.6)	11.1 (8.4-13.8)
**56-65**	43.9 (41.7-46.0)	31.1 (29.0-33.1)	12.8 (9.9-15.7)	287/488<br>55.5 (53.4-57.6)	245/613<br>38.5 (36.5-40.6)	17.0 (14.0-19.9)
**66-80**	20.5 (18.7-22.3)	10.3 (9.0-11.6)	10.2 (8.0-12.4)	150/446<br>34.1 (32.1-36.0)	92/595<br>16.6 (15.0-18.2)	17.4 (15.0-19.9)
All	**67.8 (66.6-69.1)**	**53.8 (52.6-55.0)**	**14.1 (12.3-15.8)**	**74.6 (73.3-75.8)**	**61.0 (59.9-62.1)**	**13.6 (11.9-15.2)**
