# Supplementary material for: eHealth Trends in Europe 2005-2007: A Population-Based Survey
Source: J Med Internet Res. 2008 Nov 17;10(4):e42. doi: 10.2196/jmir.1023 (PMC2629359; doi:10.2196/jmir.1023)
Supplement: Supplementary file 3 [file jmir_v10i4e42_app3.pdf]

Multimedia Appendix 3. Internet health users by age and gender

	2005			2007		
	**Men % (CI)**	**Women % (CI)**	**Diff (CI)**	**Men Frequency/N % (CI)**	**Women Frequency/N % (CI)**	**Diff (CI)**
**15-25**	60.7 (58.3-63.1)	68.4 (66.4-70.3)	-7.6 (-10.7- -4.6)	496/696 72.4 (70.1-74.6)	483/575 83.5 (81.8-85.2)	-11.1 (-13.9- -8.3)
**26-35**	58.2 (56.0-60.4)	58.8 (56.8-60.7)	-0.5 (-3.5- 2.4)	392/598 67.6 (65.4-69.7)	393/557 71.8 (70.0-73.6)	-4.3 (-7.1- -1.5)
**36-45**	50.6 (48.2-53.0)	49.4 (47.2-51.5)	1.2 (-2.0- 4.4)	392/631 60.8 (58.5-63.1)	427/679 62.8 (60.7-64.8)	-2.0 (-5.0- 1.1)
**46-55**	38.7 (36.6-40.8)	39.6 (37.6-41.7)	-1.0 (-3.9- 2.0)	284/533 52.3 (50.2-54.5)	290/611 47.3 (45.3-49.4)	5.0 (2.0-7.9)
**56-65**	27.5 (25.4-29.5)	19.3 (17.5-21.2)	8.2 (5.4- 10.9)	190/488 36.4 (34.2-38.6)	170/613 26.6 (24.6-28.6)	9.9 (6.9-12.8)
**66-80**	12.3 (10.8-13.8)	7.4 (6.2-8.5)	4.9 (3.0- 6.9)	101/446 22.6 (20.8-24.5)	55/595 9.9 (8.6-11.2)	12.8 (10.5-15.0)
**All**	**44.2 (42.7-45.8)**	**40.5 (39.2-41.7)**	**3.8 (1.8-5.8)**	**54.5 (52.9-56.0)**	**50.1(48.9-51.4)**	**4.4 (2.4-6.4)**
