# Supplementary material for: eHealth Trends in Europe 2005-2007: A Population-Based Survey
Source: J Med Internet Res. 2008 Nov 17;10(4):e42. doi: 10.2196/jmir.1023 (PMC2629359; doi:10.2196/jmir.1023)
Supplement: Supplementary file 4 [file jmir_v10i4e42_app4.pdf]

Multimedia Appendix 4. Importance of the Internet in various countries

		2005		2007		Change
	**2005/2007 (N)**	**Mean % (CI)**	**Internet compared to other media -Rank**	**Mean % (CI)**	**Internet compared to other media -Rank**	**Mean Diff % (CI**
**Germany**	974/1000	**33.7 (30.8-36.7)**	**7**	**36.8 (33.9-39.7)**	**7**	**3.1 (-1.0-7.2)**
**Denmark**	960/1021	**46.0 (42.9-49.1)**	**3**	**54.4 (51.5-57.3)**	**2**	**8.4 (4.1-12.6)**
**Greece**	1000/1000	**37.5 (34.5-40.5)**	**8**	**42.6 (39.6-45.6)**	**8**	**5.1 (0.9-9.3)**
**Portugal**	2001/1000	**51.5 (49.3-53.6)**	**7**	**55.9 (53.0-58.8)**	**7**	**4.4 (0.9-8.0)**
**Poland**	1027/1000	**41.4 (38.6-44.2)**	**7**	**48.2 (45.2-51.2)**	**6**	**6.8 (2.7-10.9)**
**Latvia**	1000/1000	**33.5 (30.7-36.3)**	**7**	**41.4 (38.6-44.2)**	**7**	**7.9 (3.9-11.9)**
**Norway**	972/1001	**38.4 (35.4-41.4)**	**6**	**48.4 (45.4-51.3)**	**4**	**10.0 (5.8-14.2)**
