# Supplementary material for: eHealth Trends in Europe 2005-2007: A Population-Based Survey
Source: J Med Internet Res. 2008 Nov 17;10(4):e42. doi: 10.2196/jmir.1023 (PMC2629359; doi:10.2196/jmir.1023)
Supplement: Supplementary file 5 [file jmir_v10i4e42_app5.pdf]

Multimedia Appendix 5. Expanded version of Table 3—Percentage of consumers who are using interactive Internet health services at least once a year

	Internet Health user			Internet user		
	2005	2007	Change	2005	2007	Change
	Mean % (CI)	frequency Mean % (CI)	Mean % (CI)	frequency Mean % (CI)	frequency Mean % (CI)	Mean % (CI)
**Self-help activities**	16.2 (15.4-17.0)	694 18.8 (17.9-19.7)	2.6 (1.4-3.8)	11.1 (10.3-11.8)	694 14.5 (13.7-15.3)	3.4 (2.3-4.5)
**Order medicine or other health products**	11.7 (11.0-12.4)	596 15.0 (14.2-15.8)	3.3 (2.2-4.4)	8.4 (7.8-9.0)	596 11.7 (11.0-12.4)	3.3 (2.3-4.3)
**Interact with Web doctor/health professional you have not met**	18.7 (17.9-19.6)	780 22.6 (21.7-23.5)	3.9 (2.6-5.1)	13.2 (12.4-14.0)	780 16.8 (16.0-17.7)	3.6 (2.5-4.8)
**Approach family doctor or other known health professional**	7.3 (6.7- 7.8)	484 12.3 (11.6-13.1)	5.1 (4.1-6.0)	5.0 (4.5-5.6)	484 9.7 (9.0-10.4)	4.7 (3.8-5.5)
**Using at least one of the interactive services above**	34.9 (33.8-35.9)	1593 42.9 (41.8-44.0)	8.0 (6.5-9.6)	24.1 (23.1-25.0)	1593 32.3 (31.3-33.4)	8.3 (6.8-9.7)
