# Supplementary material for: eHealth Trends in Europe 2005-2007: A Population-Based Survey
Source: J Med Internet Res. 2008 Nov 17;10(4):e42. doi: 10.2196/jmir.1023 (PMC2629359; doi:10.2196/jmir.1023)
Supplement: Supplementary file 6 [file jmir_v10i4e42_app6.pdf]

Multimedia Appendix 6. Total and estimated relative frequency of Internet health users and Internet users who are using at least one of the interactive services at least once a year

	All users			Internet health users			Internet users		
	**2005**	**2007**	**Change**	**2005**	**2007**	**Change**	**2005**	**2007**	**Change**
**Country**	**% (CI)**	**Observed frequency % (CI)**	**% (CI)**	**% (CI)**	**Observed frequency % (CI)**	**% (CI)**	**% (CI)**	**Observed frequency % (CI)**	**% (CI)**
**Denmark**	21.2 18.6 23.7	335/1021 32.8 29.9 35.7	11.7 7.8 15.5	33.4 (30.5-36.4)	335/731 45.3 (42.2-48.4)	11.9 (7.6-16.2)	25.3 (22.6-28.1)	335/889 36.8 (33.7-39.8)	11.4 (7.4-15.5)
**Germany**	25.1 22.4 27.7	353/1000 35.3 32.4 38.2	10.2 6.3 14.2	54.6 (51.3-57.9)	353/566 63.0 (59.8-66.2)	8.4 (3.8-13.0)	38.6 (35.4-41.8)	353/649 53.4 (50.0-56.7)	14.8 (10.2-19.4)
**Greece**	8.2 6.5 9.9	142/1000 14.2 12.1 16.3	6.0 3.4 8.7	34.0 (31.4-36.6)	142/321 48.3 (45.5-51.1)	14.3 (10.5- 18.2)	21.3 (19.0-23.6)	142/472 29.7 (26.9-32.4)	8.3 (4.8-11.9)
**Latvia**	11.0 9.1 12.9	168/1000 16.8 14.5 19.1	5.8 2.8 8.8	25.1 (22.5-27.8)	168/470 31.7 (28.8-34.6)	6.5 (2.6-10.5)	16.2 (13.9-18.5)	168/667 21.9 (19.3-24.5)	5.7 (2.2- 9.1)
**Norway**	20.7 18.1 23.3	257/1001 25.7 22.9 28.5	5.0 1.2 8.8	35.0 (32.0-38.0)	257/669 38.5 (35.3-41.6)	3.5 (-0.9-7.9)	25.5 (22.7-28.2)	257/880 28.3 (25.4-31.3)	2.9 (-1.2- 6.9)
**Poland**	15.4 13.3 17.5	229/1000 22.9 20.4 25.4	7.5 4.2 10.8	41.3 (38.5-44.1)	229/533 42.2 (39.3-45.2)	1.0 (-3.1-5.1)	28.8 (26.0-31.5)	229/667 32.7 (29.8-35.5)	3.9 (-0.1- 7.8)
**Portugal**	5.3 4.3 6.3	109/1000 10.9 9.5 12.3	5.6 3.9 7.3	20.6 (18.8-22.4)	109/383 31.3 (29.3-33.4)	10.8 (8.0-13.5)	12.8 (11.3-14.2)	109/523 23.7 (21.8-25.6)	10.9 (8.5-13.4)
