# Supplementary material for: eHealth Trends in Europe 2005-2007: A Population-Based Survey
Source: J Med Internet Res. 2008 Nov 17;10(4):e42. doi: 10.2196/jmir.1023 (PMC2629359; doi:10.2196/jmir.1023)
Supplement: Supplementary file 7 [file jmir_v10i4e42_app7.pdf]

Multimedia Appendix 7. Questionnaire in English used as basis for translation in 2007

Survey on eHealth

QTS1 Interviewer number
Interviewer number 1

QTS2 Start time
Start time 1

QTS3 Date
Date 1

QTS4 SMS id
SMS id 1

POST NOTE Postal code
Postnr.. 1

Q1 Good morning/good afternoon/good evening, my name is __________,and I am phoning from _______ on behalf of <NAME of national institution>. We are conducting a survey about sources for health information with support from the European Union and the World Health Organization (WHO). In this connection we would like to speak to <INSERT METHODOLOGY used> and who is at least 15 years old.

Everyone who takes part in the survey is completely anonymous. (We would like to emphasize that there are no right or wrong answers.) The interview will take about 8 minutes (CHECK for country-specific questions). (Your answers will be very valuable to us.) Would you be prepared to take part?

REGISTER GENDER
Male.. **1**
Female **2**

Q2 A few introductory questions.
How old are you?
Age: **1**

Q3 What is your highest level of education completed?

[Use ISCED-standard]

• BASIC SCHOOL NONE.. **01**
• BASIC SCHOOL less then 7 years **02**
• BASIC SCHOOL 8-10 grade **03**
• GENERAL UPPER SECONDARY SCHOOL **04**
• VOCATIONAL UPPER SECONDARY SCHOOL **05**
• VOCATIONAL EDUCATION AND TRAINING **06**
• SHORT-CYCLE HIGHER EDUCATION...**07**
• MEDIUM-CYCLE HIGHER EDUCATION **08**
• BACHELOR **09**
• LONG-CYCLE HIGHER EDUCATION **10**
• VERY LONG-CYCLE HIGHER EDUCATION **11**
• Do not want to answer **12**

Q4 How many children under the age of 18 are living in your household?
Number of children: ....

Q5 Where do you live?
• City (main cities) **1**
• Minor cities (suburbs/vicinity to larger cities) **2**
• Villages **3**
• Rural area (country-side, scattered population).. **4**

Q6 Which of these descriptions best describes your situation or applies to what you have been doing for the last month:
READ OUT

• Paid work (including self-employed) -> 6b...1
• In education 2
• Unemployed 3
• Permanently sick or disabled 4
• Retired.. 5
• In community or military service 6
• Housework, looking after children or other persons (e.g. maternity leave) 7
• (Other) 8
• (Don't know) 9

Filter: Q6=1

Q6B What is your job?

DON'T READ OUT (TWO OPTIONS POSSIBLE FOR HEALTH PERSONNEL)

White collar group

• Manager, head of other people 1
• Health personnel, e.g. has treatment responsibility, higher education, licence or authorization such as medical doctor, psychologist, nurse, physiotherapist............... 2
• Other, e.g. lawyer, consultant, secretary, artist, researcher, educationalist, kindergarten teacher.. 3

Blue collar group

• Health care assistants, e.g. with no formal treatment responsibility, short or no formal training, no licence or authorization... 4
• Skilled workers (formal full-time schooling for at least a year or equivalent), e.g. manual/routine work, trained plumber, carpenter, mechanic, cook........................5
• Unskilled or semi skilled workers, with no or short time of formal training, e.g. manual/routine worker, cleaning personnel, drivers, kindergarten assistant........ 6

Q7 I will now read a list of various sources of information about health or illness, and would like to know how important these are to you. Please would you answer on a scale from 1 to 5, where 1 is ¨not important¨ and 5 is ¨important¨.

READ OUT

1 Not important 2 3 4 5 Important

• Internet **1 2 3 4 5 6** 1
• TV/radio **1 2 3 4 5 6** 2
• Books, medical encyclopaedias and leaflets **1 2 3 4 5 6** 3
• Courses and lectures **1 2 3 4 5 6** 4
• Newspapers, magazines **1 2 3 4 5 6** 5
• Family, friends and colleagues **1 2 3 4 5 6** 6
• Pharmacies **1 2 3 4 5 6** 7
• Direct face-to-face contact with health professionals **1 2 3 4 5 6** 8

Q8 How often do you use the Internet?

READ OUT

• Every day 1
• Every week 2
• Every month 3
• Less than once a month 4
• I have never used the Internet **(⇒ Q17)** 5
• I have never used it, but I have asked others to use it for me**(⇒ Q8B)** 6

Filter: Q8=6

Q9 How often do you use the Internet to get information about health or illness?

READ OUT

• Every day 1
• Every week... 2
• Every month 3
• Every six months 4
• Every year 5
• Less than once a year 6
• Never**(⇒ Q17)** 7

Q10 I will now read out some purposes for which the Internet can be used to provide information related to health or illness, and would like to know how often you use the Internet for these purposes.
How often do you use the Internet to:

REPEAT SCALE WHEN NECESSARY
Every day
Every week
Every month
Every six months
Every year
Less than once a year
Never

• interact with health professionals you have not met face-to-face **1 2 3 4 5 6 7** 1
• participate in forums or self help groups(focusing on health or illness) **1 2 3 4 5 6 7** 2
• order medicines or other products related to
health or illness management online **1 2 3 4 5 6 7** 3
• read about health and illness **1 2 3 4 5 6 7** 4

Q10B I will now read out some purposes for which the Internet can be used to provide information related to health or illness, and would like to know how often you use the Internet for these purposes.
Do you, always, often, sometimes, rarely or never, use the Internet to. . . .

REPEAT SCALE WHEN NECESSARY
Always – Often – Sometimes - Rarely – Never

• find health information that can help you decide whether to
consult a health professional **1 2 3 4 5** 1
• find health information prior to an appointment **1 2 3 4 5** 2
• find information after an appointment with health professionals
(e.g. for second opinion) **1 2 3 4 5** 3

Q11 Have you approached your family doctor, specialist, or other health professional(s) over the Internet (web or e-mail), e.g. read their website, request or renew prescription, schedule an appointment, ask particular health questions or read your health record?
• Yes(⇒ **Q13**) **1**
• No(⇒ **Q14**) **2**
Filter: Q12=1

Q12
In which connection and for what purposes have you approached your family doctor, specialist, or other health professional(s) via the Internet?
READ OUT.
CHECK AS MANY AS APPLY.
I
• Request or renew prescription via e-mail or web..**1**
• Schedule an appointment **2**
• Ask particular health questions **3**
• Access to read your patient record **4**
• Read their web site **5**
• Other **6**
• DO NOT READ OUT
Do not know **7**
• DO NOT READ OUT
Do not want to answer **8**
Filter: Q12=2

Q13
There are different reasons for not approaching your family doctor, specialist or other health professional(s) via the Internet.
Which reasons apply for you?
READ OUT.
CHECK AS MANY AS APPLY.

• I worry about confidentiality **1**
• I prefer face-to-face communication **2**
• My family doctor or specialist do not offer such services **3**
• I have not needed to contact them **4**
• Other **5**
• DO NOT READ OUT
Do not know **6**

• DO NOT READ OUT
Do not want to answer **7**

Q14

When evaluating an Internet health site, how important are the following factors?
Please would you answer on a scale from 1 not important to 5 important.
READ OUT
1 Not important 2 3 4 5 Important

• Secure handling of personal information **1 2 3 4 5 6** 1
• Information in my own language **1 2 3 4 5 6** 2
• Updated information **1 2 3 4 5 6** 3
• Interactivity, e.g. Question-and-answerservice, discussion groups, chat **1 2 3 4 5 6** 4
• Health professionals are involved **1 2 3 4 5 6** 5
• Clearly stated who is responsible for orsponsors the site **1 2 3 4 5 6** 6

Q15

Has information on health or illness which you have obtained from the Internet led to any of the following?
READ OUT
Yes No Do not know

• Feelings of anxiety **1 2 3** 1
• Feelings of reassurance or relief **1 2 3** 2
• willingness to change diet or other lifestyle habits **1 2 3** 3
• suggestions or queries on diagnosis or treatment to your family doctor,
Specialist or other health professionals **1 2 3** 4
• Changing of use of medicine without consulting your family doctor,
specialist or other health professionals **1 2 3** 5
• Making, cancelling or changing an appointment with your family doctor,
specialist or other health professionals **1 2 3** 6

Q16 **We would now like to ask you a few questions about what you believe you will do in the future.**

Given that you were provided the possibility, state how likely it is that you will do the following during the next year?
Please would you answer on a scale from 1 unlikely to 5 very likely.

1 Unlikely 2 3 4 5 Very likely 6 Do not know / not applicable

• Look for information about health or a
particular illness on the Internet **1 2 3 4 5 6** 1
• Participate in forums or self-help groups focusing on
health or illness online **1 2 3 4 5 6** 2
• Order medicines or other health products online **1 2 3 4 5 6** 3
• Have consultations with health professionals online **1 2 3 4 5 6** 4
• Make, cancel or change an appointment with your family doctor,
specialist or other health professionals online **1 2 3 4 5 6** 5

Q17 **If you were to find a new doctor, state the importance of the following factors for your decision.**

Please would you answer on a scale from 1 not important to 5 important.
1 Not important 2 3 4 5 Important 6 Do not know / not applicable

• The possibility to request or renew prescriptions via e-mail or web **1 2 3 4 5 6** 1
• The cost of services **1 2 3 4 5 6** 2
• The possibility to schedule or change appointments online **1 2 3 4 5 6** 3
• Information on the doctor’s practice, e.g. waiting lists or scores
on public evaluation **1 2 3 4 5 6** 4
• That the office has its own website **1 2 3 4 5 6** 5
• Recommendation by others **1 2 3 4 5 6** 6
• The possibility to communicate by e-mail **1 2 3 4 5 6** 7
• The possibility to get reminders by SMS text message **1 2 3 4 5 6** 8
• Online access to read your electronic patient record **1 2 3 4 5 6** 9
• Accessibility, such as nearby office and convenient opening hours **1 2 3 4 5 6** 10

Q18

To end off, a few background questions. How many times did you visit a doctor during the last 12 months? (Include hospitalisation or visits to the outpatient department; do NOT include visits to the dentist).

Do not know : type 98
Do not want to answer :type 99

Number of times:... 1

Q19

Are you, or someone close to you, currently experiencing long-term illness or disability?

CHECK AS MANY AS APPLY

• Yes, I am **1**
• Yes, someone close to me is **2**
• No **3**
• DO NOT READ OUT - Do not know **4**
• DO NOT READ OUT - Do not want to answer **5**

Q20

How would you assess your present state of health?
READ OUT
• Very good. **1**
• Good **2**
• Fair **3**
• Bad **4**
• Very bad **5**
• DO NOT READ OUT - Do not know **6**
• DO NOT READ OUT - Do not want to answer **7**

Filter: Q10 = 6,5,4,3,2 or 1

Q21

Did you go online to obtain information on either of the following during the last six months?
CHECK AS MANY AS APPLY

READ OUT
• information on life style, e.g. nutrition, diet, exercise, stop smoking. **1**
• information on pregnancy or baby-care **2**
• information on managing aspects of illness (such as health insurance,
rights to sick-leave, public assistance etc.) **3**
• information on a specific illness **4**

Filter: Q21 =4

Q21B

Would you characterize this illness as
• very severe **1**
• severe **2**
• not severe **3**

Thank you very much for your help!
